# Cooperative Power-Domain NOMA Systems: An Overview

**DOI:** 10.3390/s22249652

**Published:** 2022-12-09

**Authors:** Mujtaba Ghous, Ahmad Kamal Hassan, Ziaul Haq Abbas, Ghulam Abbas, Aseel Hussien, Thar Baker

**Affiliations:** 1 Telecommunication and Networking Research Lab, GIK Institute of Engineering Sciences and Technology, Topi 23640, Pakistan; 2 Faculty of Electrical Engineering, GIK Institute of Engineering Sciences and Technology, Topi 23640, Pakistan; 3 Faculty of Computer Sciences and Engineering, GIK Institute of Engineering Sciences and Technology, Topi 23640, Pakistan; 4 College of Engineering, University of Sharjah, Sharjah 27272, United Arab Emirates; 5 College of Computing and Informatics, University of Sharjah, Sharjah 27272, United Arab Emirates

**Keywords:** cooperative communication, successive interference cancellation, power-domain NOMA, precoder, equaliser, antenna diversity, outage probability

## Abstract

Interference has been a key roadblock against the effectively deployment of applications for end-users in wireless networks including fifth-generation (5G) and beyond fifth-generation (B5G) networks. Protocols and standards for various communication types have been established and utilised by the community in the last few years. However, interference remains a key challenge, preventing end-users from receiving the quality of service (QoS) expected for many 5G applications. The increased need for better data rates and more exposure to multimedia information lead to a non-orthogonal multiple access (NOMA) scheme that aims to enhance spectral efficiency and link additional applications employing successive interference cancellation and superposition coding mechanisms. Recent work suggests that the NOMA scheme performs better when combined with suitable wireless technologies specifically by incorporating antenna diversity including massive multiple-input multiple-output architecture, data rate fairness, energy efficiency, cooperative relaying, beamforming and equalization, network coding, and space–time coding. In this paper, we discuss several cooperative NOMA systems operating under the decode-and-forward and amplify-and-forward protocols. The paper provides an overview of power-domain NOMA-based cooperative communication, and also provides an outlook of future research directions of this area.

## 1. Introduction

To make fifth-generation (5G) networks more efficient and beyond fifth-generation (B5G) networks a reality, recently a lot of cutting-edge concepts have been proposed and examined. Small cells, device-centric architecture, beamforming, full-duplex technology, massive multiple-input multiple-output (MIMO), millimeter (mm) and terahertz (THz) waves, non-orthogonal multiple access (NOMA), and reconfigurable intelligent surface (RIS) are some of the major enabling technologies that have been taken into account for 5G and B5G systems. The bandwidth of millimeter waves can be ten times greater than that of the entire 4G cellular band [1]. The idea of ultra-massive MIMO (UM-MIMO), which uses plasmonic materials for transceiver construction to achieve THz band capacity, has emerged [2]. In B5G IoT, various resources of the vertical industries will require the ability to support a huge number of connections, motivating fundamental research on how to increase the system’s spectral efficiency. In this concern, one of the key enabling technologies is NOMA [3]. Additionally, RIS-assisted NOMA is further considered to be a promising new technology that can reconfigure the surroundings for wireless transmission utilising software-controlled reflection to fulfill the needs of 5G and B5G communication [4].

**Figure 1:**
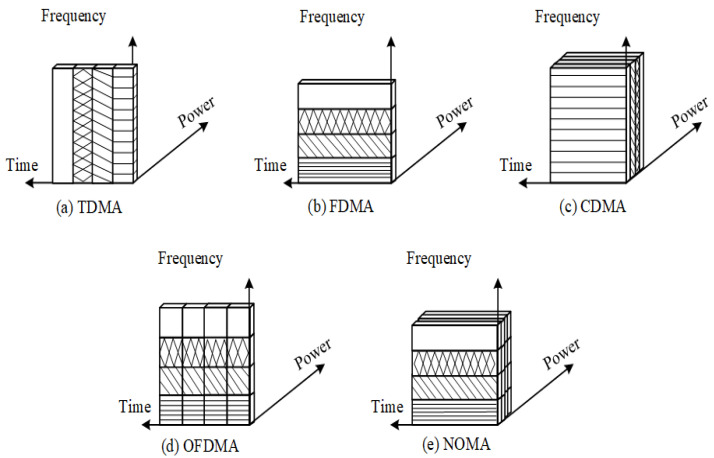
Pictorial description of different multiple access schemes, i.e., (**a**) TDMA, (**b**) FDMA, (**c**) CDMA, (**d**) OFDMA, and (**e**) NOMA.

**Figure 2:**
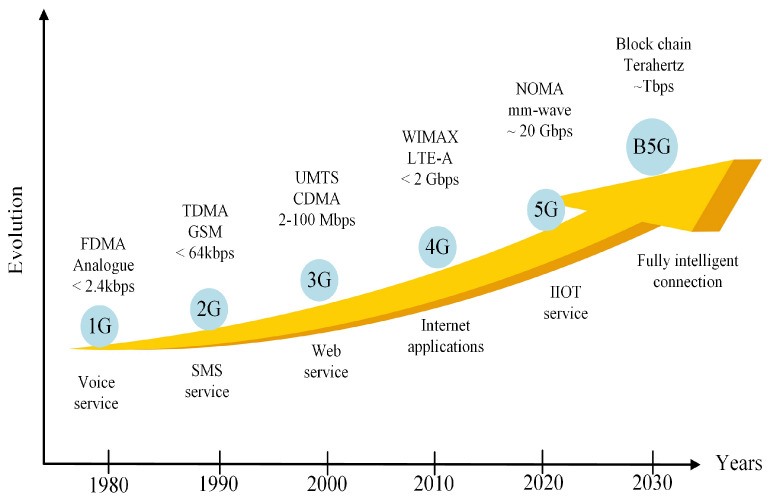
Evolution of mobile wireless systems from 1G to B5G along with multiple access techniques used.

In the last few decades, mobile communication technology has progressed from the audio-centric first generation (1G) to the data-centric recent generations to cater for wide range of applications. In different generation of mobile communication, MA methods permit users to utilise the time and/or spectrum resource simultaneously, and this may be conducted without compromising the users’ quality-of-service (QoS). In Figure 1, MA techniques such as frequency-division multiple access (FDMA), time-division multiple access (TDMA), orthogonal frequency-division multiple access (OFDMA), code-division multiple access (CDMA), and non-orthogonal multiple access (NOMA) show how various users (represented by different line designs) are assigned spectra. The analogue 1G system, for instance, uses FDMA to enable voice communication only [5]. By using FDMA [6], frequency bands are separated into distinct channels and hence the channels are then assigned to different users in 1G. TDMA became increasingly feasible with progress in digital communication systems, and it is used in the second-generation (2G) systems for text and voice applications [7]. In TDMA, each user is authorised to transmit on the same frequency band, but only within a specified time period. The third-generation (3G) systems use CDMA to offer video, voice, and data services [8]. CDMA [6] provides consistent bandwidth and transmit power where users can send data at the same time utilising the same frequency range, employing pseudo-code of sorts. To enable rich multimedia broadband services that desire low latency and high data rates, the fourth-generation (4G) long-term evolution-advanced (LTE-A) [9] was introduced, which uses OFDMA and single-carrier FDMA for downlink and uplink, respectively. In OFDMA [10], MA is achieved by distributing portions of subcarriers to each individual. 4G technologies included carrier aggregation (CA), heterogeneous network (HetNet) deployment, coordinated multi-point (CoMP), improved broadcasting and multi-casting services, and full-dimension multiple-input multiple-output (FD MIMO) [11]. The peak data throughput requirements for LTE-A are 1 Gbps for downlink, with 5 ms latency and a mobility of up to 350 km/h [12]. However, sophisticated albeit important future applications such as tactile communication, augmented and virtual reality, ultra high-definition video quality, and online remote control monitoring are some of the challenging performance objectives. Hence, these aspects generated the need for 5G and B5G systems [13]. The evolution of mobile wireless systems with multiple access technologies is discussed in [14] and depicted in Figure 2. The 5G and B5G technologies are able to provide solutions for different and mission-critical applications, such as massive machine type communication (mMTC) [15], enhanced mobile broadband (eMBB) [16], and ultra-reliability and low latency communication (URLLC) [17], as per International Telecommunication Union (ITU) [18]. The first commercial version of a 5G system based on 3G Partnership Project (3GPP) release has 15 standards. The new generation of networks is likely to have additional improvements to enable advanced services and applications [19].

Because of the scarcity of wireless spectrum resources, desired system capacity remains a major issue for the future mobile communication generations. By superposing the signals of multiple users in the same orthogonal resource block (RB), NOMA is recognized to be an effective method for supporting the needs of large-scale access [20]. In addition, in MIMO with a larger antenna order, and its special case, i.e., multiple-input single-output (MISO) could achieve a higher capacity. The MIMO-NOMA systems can take advantage of the extent in both the space and power domains, thus enhancing system spectrum efficiency even more.

### 1.1. Scope

Most studies in the literature have emphasised on code-domain NOMA (CD-NOMA) [21,22,23,24,25], or power-domain NOMA (PD-NOMA) [22,26], albeit without cooperation between users or devices. However, to the best of our knowledge, no comprehensive survey or an overview has taken into account a machine learning-based PD-NOMA, RIS-based PD-NOMA, and transmit antenna selection-based PD-NOMA systems. Hence, the present survey includes a majority of NOMA variants within the broad category of PD-NOMA and cooperative communication in PD-NOMA.

### 1.2. Motivation and Contributions

The demand for data rate and capacity has increased dramatically due to the change in users’ preferences from voice messages to video streaming, and downloading multimedia applications. For example, video streaming in 4K/8K high definition requires a data rate of about 300 Mbps, which strains the present networks’ capacity when a large number of user devices are provided. Hence, there is motivation for the utilization of NOMA in next-generation mobile networks. Cooperative NOMA is possibly the best technique for radio access that improves spectrum efficiency, transmission rate, and latency [27]. Furthermore, NOMA surpasses the present OMA system in terms of total throughput and a performance boost is predicted with the adoption of an improved power allocation technique [28]. However, further research is needed. It was suggested that NOMA is the prime technology to be introduced in the new communication systems [29,30,31,32,33]. Furthermore, resource allocation has been identified as a critical approach for maximising a number of network performance metrics, e.g., to maximise the sum rate among other key performance indicators (KPIs). Resource allocation in the NOMA system, in particular, has attracted tremendous attention in the literature because it can produce considerable performance gains over the OMA system [34]. However, some of the research in [34] investigated an improvement in the performance of users with bad channel conditions through the integration of NOMA by optimising the resource allocation in MIMO-NOMA networks.

A few difficulties that represent open study areas for cooperative NOMA techniques have been addressed in our survey paper. It is worth noting that a some prior articles have offered a similar notion with different issues and approaches. Multiple review articles have provided detailed surveys on PD-NOMA [35,36,37,38,39]. The authors of [35] covered aspects of this novel technology, from its use in association with MIMO technologies through NOMA and the connection between cognitive radio and NOMA. The authors also examined the state of standardization initiatives for NOMA deployment in LTE and 5G networks. The authors of [36] examined the analysis of capacity, power allocation algorithms, user-pairing schemes, and user fairness in NOMA, as well as the latest advancements of NOMA in 5G networks. Their research also looked at how NOMA works when combined with other well-known techniques for communication such as MIMO, space-time and network coding, and beamforming. Similarly, the authors of review article [37] explained NOMA concepts, important characteristics, advantages and disadvantages, and then compared these features including the spectrum efficiency and complexity of the receiver. Power domain was explained in [38], where the authors discussed two well-known categories of NOMA, i.e., PD-NOMA and CD-NOMA. Moreover, a comparison of the methodology of different NOMA schemes in terms of complexity of the receiver due to successive interference cancellation (SIC) was discussed as well. The authors of [39] presented a comprehensive overview of multiple-access systems, methodologies, and techniques for NOMA optimization. They also reviewed the literature’s taxonomy of multiple-access schemes, followed by a full description of NOMA’s aims, limits, challenges, and solutions. The decoding algorithms and KPIs utilized in NOMA were also discussed in this work. Our paper, on the other hand, concentrates on cooperative PD-NOMA networks. It is worth noting that only a few studies have looked into NOMA-based cooperative communication to date, which indicates that research in this area is still in its early stages. Most of the literature on the spectrum efficiency and system capacity is addressed in the indicated efforts on cooperative communication. There have not been many studies published on the system’s user-fairness, which suggests that an important aspects of B5G networks is to assure increased access of network resources for both far and near users. Besides, the opportunities and challenges associated with cooperative power-domain NOMA and integrating RISs with NOMA in the envisioned next-generation mobile networks are thoroughly covered in this paper. The list of surveys [23,26,40,41,42,43], that have appeared on PD-NOMA schemes is compared with our work in Table 1. Specifically, we will focus on the following features.

1. The current literature on cooperative PD-NOMA is extensively reviewed to provide a better picture of the research that has been conducted in this field. In this paper, we discuss a thorough, up-to-date review of the integration of PD-NOMA with antenna selection schemes, MIMO systems, mmWave, CoMP, cognitive radio, THz bands, cooperative communications, SWIPT, transmit antenna selection, beamforming, clustered-based NOMA, and the other modern communications techniques, in order to improve the systems’ overall rates and, consequently, the spectral efficiency in upcoming wireless networks.
2. An overview of the existing NOMA systems is presented. Furthermore, cooperative PD-NOMA, the concept, benefits, problems, and applications are outlined, and parallels are drawn with the well-established cooperative networks and the rationale for integrating cooperative approaches with PD-NOMA is provided. A qualitative analysis is conducted to establish the performance gain of PD-NOMA over OMA-based cooperative networks in particular.
3. The fundamentals of communication strategies under consideration (RIS-assisted NOMA) are reviewed. The main concept of RISs, namely the reconfiguration of the users’ propagation environment, is demonstrated, and a description of NOMA’s capacity to promote the sharing of spectra between mobile users to maximise spectral efficiency is illustrated. This survey also covers RIS-NOMA security provisioning. The influence of using a RIS with security is highlighted by studying the RIS-NOMA applications to improve physical layer security with regard to passive eavesdropping. The use of RIS-NOMA to cover cooperative PD-NOMA communications is then investigated.
4. The impact of machine learning on NOMA-based schemes is thoroughly investigated, and the directions for further research on NOMA with ML support are described.
5. In the end, we identify the open research challenges and prospective research directions which may enable the researchers to contribute some effective results in the aforementioned domains.

### 1.3. Organization of the Paper

**Figure 3:**
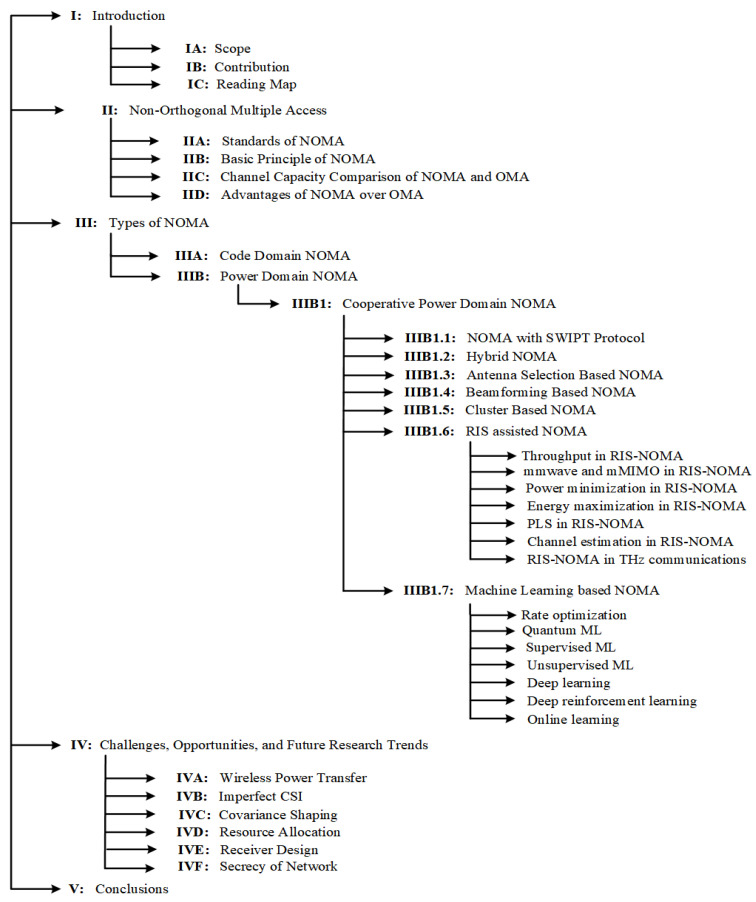
Road map of the paper.

The paper’s structure is provided in Figure 3. All acronyms used in this study are listed in Table 1. The remaining portion of the paper is organised as follows. The NOMA’s historical perspective is addressed in Section 2. The cooperative PD-NOMA variations of 5G are examined in Section 3. The outstanding concerns and research difficulties of NOMA are reviewed in Section 4, and the study concludes in Section 5.

## 2. Non-Orthogonal Multiple Access

Several MA schemes are presented in the introduction section; however, there exist multiple constraints and trade-offs in any particular MA scheme. For brevity, let us investigate only two such constraints. First, the number of users served at the same time is limited. Second, user scheduling and robust feedback mechanisms are necessary to ensure orthogonality. These constraints are overcome by NOMA, which is a superior resource allocation approach to OMA as it has the ability to maximise system’s spectral efficiency. The basic concept and brief mechanism of NOMA is discussed next.

### 2.1. Standards of NOMA

The integration of NOMA with enhanced mobile broadband (eMBB) improves the data rate fairness, capacity, and QoS between users in ultra-dense networks as discussed in [44,45,46]. NOMA, on the other hand, resolves the huge connectivity and vast coverage area requirements for URLLC and massive machine type communication (mMTC). If the transmission is grant-free, it delivers better quality of link with minimal latency. Furthermore, under LTE Release-13, the 3GPP introduced multi-user superimposed transmission (MUST) as an initial standard for downlink (DL) applications. The 3GPP classifies MUST system into three main groups based on DL scenarios: MUST 1, MUST 2, and MUST 3. In MUST 1, the constellation symbols are constructed without dependence on the grey mapping by mapping two users bits separately. Grey mapping is used in MUST 2 to combine the user’s bits to generate constellation symbols. MUST 3 uses the grey mapping, but the power ratio of signal is not applicable in this standard.

### 2.2. Basic Principle of NOMA

It is commonly accepted, e.g., in [47,48,49,50] that to separate user information, NOMA uses the successive interference cancellation (SIC) method at the receiver’s end. During the SIC process, however, information from users with lower channel gains is extracted by users with higher channel gains.

#### 2.2.1. Superposition Coding (SC)

Multiple users’ signals are mapped in the power domain and at the transmitter utilising SC in NOMA. Capacity on a scalar Gaussian broadcast channel is achieved by combining user information into a single-signal source by using a non-orthogonal SC technique [36].

**Figure 4:**
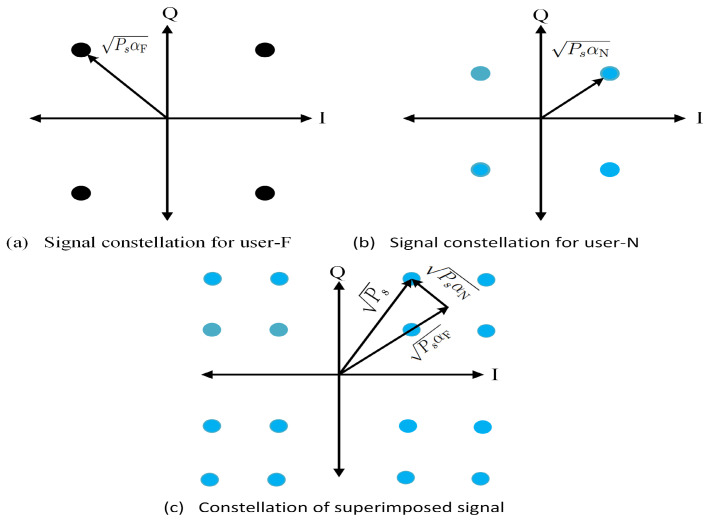
Hierarchical constellation diagram of superposition coding example.

Consider a situation in which K={user-N,user-F} represents the total number of users where user-N denotes the near user and user-F represents the far-user. Complex-valued symbols, $x_{N}$ and $x_{F}$ for user-N and user-F, respectively, are mapped into their appropriate M-ary modulation constellation with total transmitted power $P_{s}$ and the power allocation coefficients $\alpha_{N}$ and $\alpha_{F}$ for user-N and user-F, respectively. For this example, both users will utilise quadrature phase-shift keying (QPSK), as shown in Figure 4. In SC, user-F’s (higher transmit power) constellation is superimposed on user-N’s (less power) constellation to produce a mapped constellation, as shown in Figure 4c.

#### 2.2.2. Successive Interference Cancellation

**Figure 5:**
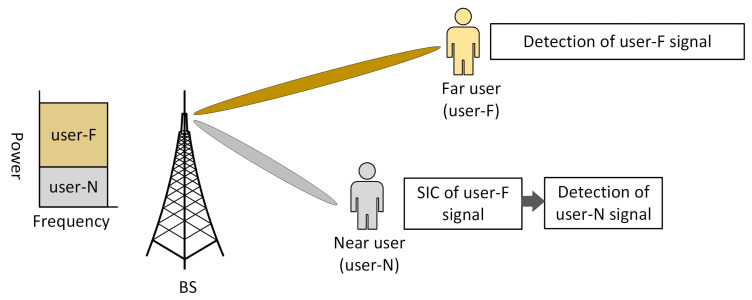
Representation of a conventional two-user NOMA system where user-N assists user-F.

The mapped signal is then broadcast across the same time and frequency resources as the original signal. The required signals are detected at the receiver using the SIC detection approach, which is possible by providing substantial power allocation differences between users. The CWIC and SLIC are the two types of interference cancellation explored in NOMA. To keep CWIC’s performance closer to that of ideal SIC, its power allocation factor must be more than or equal to 0.65 for the user having bad channel conditions [51]. An CWIC receiver is able to reduce the interference between users. SLIC receivers, on the other hand, decreases the complexity of NOMA receivers, but suffers significant performance loss if the weak user’s power allocation factor is not high enough. The SIC process of a conventional NOMA system is shown in Figure 5.

### 2.3. Advanced Channel Coding and Modulation

Channel coding and modulation, which are essential physical layer technologies, offer effective means for a radio link that must work close to its channel capacity while maintaining appropriate signal waveforms of baseband and RF at transmitters and/or receivers. In general, transmit waveforms, multiple accesses, and ans similar techniques are all included in channel coding and modulation.

#### 2.3.1. Channel Coding

A binary channel can be binary erase, symmetric, or additive white Gaussian noise. The single-link channels’ Shannon limit serves as the performance benchmark. As multiple access techniques are primarily orthogonal, they have been important in mobile communications since the 2G era, in situations where multiple users serving from same base station or spatial domains use separate frequency and time resources. NOMA, however, can be utilised in addition to OMA to expand system capacity and service additional connections. This opens up a completely new field called “multi-user oriented channel coding”, which is essentially an improved form of “interleave-division multiple access” (IDMA). It is important since multi-user channel channel capacity is not yet fully understood or established, particularly for uplink with the near-far effect and independent channel fading. Theoretically, any channel code might be taken into consideration in order to boost the capacity of multi-user channels, provided that the code itself can allow for optimization, such as going from a single user to multiple users. Multi-user low-density parity-check codes (LDPC) have recently been suggested for non-orthogonal uplink transmission [52].

Non-binary (sometimes referred to as multi-variant) codes can be taken into consideration to improve the channel codes’ robustness in fading channels and under high SNR conditions. Multi-variant codes currently come in two main categories. The multi-variant LDPCs, as discussed in [53] are classified in the Galois field (q). Lattice codes are the second class of these codes. The low-density lattice code [54], which is also represented by the Tanner graph and parity check matrix, is a very promising variation of the parent code.

#### 2.3.2. Modulation and Spreading

The point distribution of the QAM constellation is widely known to be not at all Gaussian. Although QAM’s generation and its demodulation are easier than those of many other modulations, it is not the best option in terms of capacity. Satellite communications and a variety of broadcasting networks have both employed amplitude-phase shift keying (APSK). APSK is particularly resistant to the power amplifier’s non-linearity. In comparison to other modulation techniques, APSK can tolerate more phase noise. As a result, APSK might be used for high-frequency communications at THz and deep mmWave frequencies.

Modulation and channel coding can sometimes be implemented together, as in the case of Trellis codes. When modulation and channel coding are created separately and detection and decoding are conducted individually, information loss between the two can be minimised by using joint coding and modulation that is well designed. When SNR is high, the performance boost is more obvious. One crucial factor is receiver complexity, which should be kept under control, i.e., not significantly higher than that of the conventional receiver. Faster-than-Nyquist (FTN) [55] signalling is a suitable contender for an extremely high SNR operating point and raises spectral efficiency. However, it has the drawback of introducing inter-symbol interference (ISI), which must be muted or eliminated. FTN is comparable to non-orthogonal transmission in this regard. NOMA can leverage symbol-level spreading [56] to support numerous concurrent users on the network.

### 2.4. Benefits of NOMA over OMA

The merits of NOMA over OMA are discussed as follows.

- **High spectral efficiency**: NOMA has a higher SE when compared with OMA, because one RB offers services to many users. In OMA systems, each user is allotted by one RB, resulting in bandwidth loss [57]. NOMA may also be readily discussed as mMIMO, mmWave, HetNets, and device-to-device (D2D) systems to further boost network throughput.
- **Massive connectivity**: Because of its non-orthogonal features, NOMA has the capability to incorporate high plurality of smart devices. Having smaller and irregular size of packets, it is suited for both the tactile internet [58] and the IoT [59].
- **Fairness**: NOMA ensures user fairness by assigning a higher power to weak users (those with poor channel conditions) and a smaller amount to strong users. Then, in terms of throughput, stronger and weaker users are assured QoS.
- **Ultra low latency**: Due to HetNet design of B5G networks, latency requirements are more demanding. Because OMA methods rely on access-grant requests, which increases transmission delay and signalling cost, they are not appropriate for such architectures. To overcome this problem, NOMA is utilised which allows for grant-free transmission, which is highly useful in the uplink scenario. Furthermore, NOMA allows for variable scheduling of many devices based on the application and QoS requirements.

## 3. Types of NOMA

**Figure 6:**
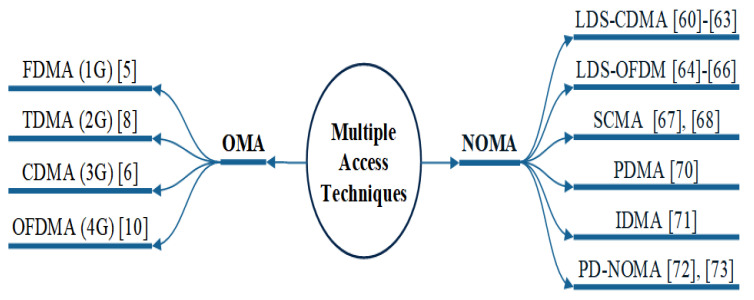
Multiple access techniques: types of NOMA and OMA.

NOMA is emerging as a key enabler for future-generation wireless networks, and hence many forms have been proposed and studied. Multiple users broadcast information on shared resources in all NOMA schemes, and multiple users can use the same physical channel, allowing for significant capacity gains. The receiver adopts a joint detection method such as SIC or message passing algorithm (MPA) to identify non-orthogonal signals. While the fundamental ideas of all NOMA schemes are the same, they are divided by the method of how non-orthogonality is accomplished. An overview of MA techniques is depicted in Figure 6.

### 3.1. Code Domain NOMA

*Low-density spreading code-division multiple access (LDS-CDMA)*: LDS-CDMA is based on the original CDMA principle which is meant to reduce the interference in traditional CDMA systems by utilising LDS instead of traditional spreading sequences. LDS-CDMA’s essential idea has been described in [60,61]. Furthermore, Refs. [60,62] discussed an iterative multi-user detection (MUD) based an MPA that is less complicated than the optimum detector. The authors in [63] proposed a systematic method for developing LDS codes for LDS-CDMA, with the fundamental concept of mapping a spreading matrix containing the spreading sequences of constellation signature. In addition, an information theoretical analysis was used to compute the region of capacity in [61].

*Low-density spreading-aided OFDM (LDS-OFDM)*: OFDM and multi-carrier (MC)-CDMA have many similarities, when it comes to spreading in frequency-domain, which distributes users’ symbols between all OFDM subcarriers. If a chip’s quantity of codes is equal to the subcarriers’ quantity. Then, across all subcarriers, numerous mobile equipment may be accommodated by overlaying the distinct, spreading sequences for the specific users on top of one another. The authors of [24] presented the LDS-OFDM system’s concept and characteristics, including its frequency variation order, receiver architecture, and capacity to function under rank-deficient situations with more users than chips. In order to manage receiver complexity, the authors of [64] set a limitation on the numbers of users for each subcarrier. In addition, in [65,66], LDS-OFDM was compared to both OFDMA and single-carrier (SC)-FDMA in order to evaluate the peak-to-average-power ratio (PAPR), with system and link-level performance.

*Sparse code multiple access (SCMA)*: Another significant NOMA strategy is the recently suggested SCMA technology, which is based on code-domain multiplexing derived from the fundamental LDS-CDMA scheme. SCMA was thoroughly explored through the multiplexing features, transmission features, factor graph representation, and message-passing algorithm-based receiver design. The message-passing method, which is similar to LDS-CDMA, may also be utilised by the receiver of SCMA for MUD. The receiver complexity, on the other hand, may become extreme. Improved variations of the message forwarding method have been developed in [67,68] to overcome this issue.The authors of [67] presented an alternate search method that depends on traditional signal entropy theory for removing all redundant conditional channel probability calculations while maintaining decoding speed. In contrast, Ref. [69] proposed a simple combination of repeated decoder for achieving a convincing balance between complexity and performance. On the other hand, the strong turbo-principle has been introduced in [68] for enabling communication between the decoder of the channel and SCMA detector in order to increase the BER performance of SCMA.

*Pattern-division multiple access (PDMA)*: In this technique, multiplexing may be performed in the code, spatial, and power domains [70]. Non-orthogonal patterns are used at the transmitter to minimise user overlaps. The spread of multiple users is defined by the pattern matrix of the subcarrier. MPA can be modified at the receiver to detect the distributed information, and MPA-SIC is used by multiplexed users in both the space and power domains.

*Interleave-division multiple-access (IDMA)*: Inter-leavers differentiate distinct users in IDMA [71]. The interleaver requires additional bandwidth and memory resources at the receiver and the transmitter. To achieve better performance, an elementary signal estimator is utilised at the receiver. Multiplexing is performed in the power domain in PD-NOMA. At the transmitter, signals from various users are superimposed by assigning the user’s power with identical subcarriers, and the succeeding signal is transmitted.

The kinds of NOMA described above are summarized in Table 2.

### 3.2. Power Domain NOMA

**Figure 7:**
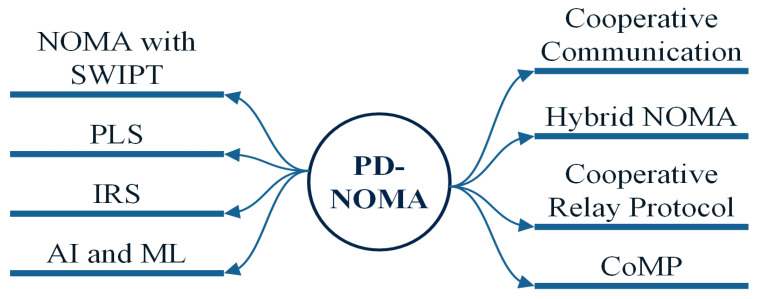
Different power-domain NOMA versions employed in 5G.

Different PD-NOMA versions employed in 5G as shown in Figure 7 are discussed in this section. PD-NOMA is a method for simultaneously serving many users by differentiating power levels. This method is most commonly used in 5G to enhance energy efficiency, spectral efficiency, and latency. The subsections that follow provide a thorough explanation of each PD-NOMA variation.

#### 3.2.1. Cooperative Relaying Communication

**Figure 8:**
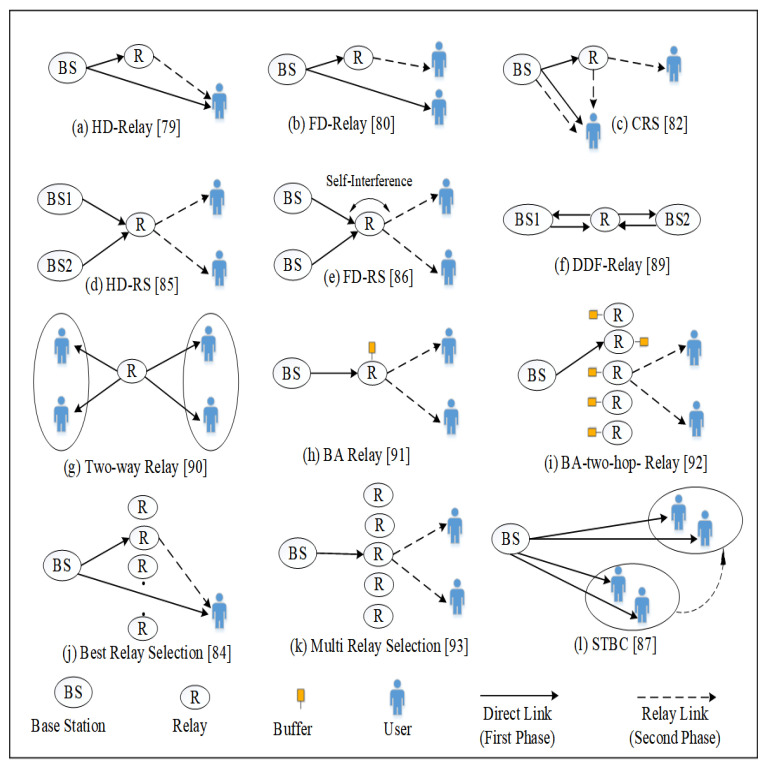
Cooperative communication to enhance the networks spectral efficiency.

To achieve better signal strength between the BS and destination, cooperative communication employs one or more relays. It employs two time frames: direct phase transmission is used in the first frame and forwarding information from relays are relayed to ultimate destinations in the second frame, as illustrated in Figure 8. It has numerous benefits, listed as: (i) it expands the coverage area, (ii) it reduces the multi-path fading effect, (iii) it increases system capacity, (iv) it eliminates the difficulty of antenna terminals mounting process, and (v) QoS is enhanced for cell edge users.

Relays employ DF and/or AF protocols to send data from one end (transmitter) to the other end (receiver) in this technique. These are categorised as either half-duplex (HD) or full-duplex (FD) systems depending on the relaying process. Researchers have utilised NOMA with cooperative communication to enhance the networks’ spectral efficiency. Reduced redundancy in systems, fairness, and increased weak users diversity gain are all advantages of this integration. Moreover, many cooperative NOMA versions are explored next, based on the aforementioned benefits.

*Cooperative relaying NOMA (CR-NOMA)*: Recent work has investigated the diversity gain in order to make use of the NOMA technique’s existing knowledge [72]. In this approach, a user with good channel conditions decodes other users’ messages and works as a relay to facilitate the weak users (with bad channel conditions) for better reliability of reception. It increases the gain in diversity for all users. Nonetheless, it has been found that during the cooperative phase, when data are transferred using relay towards weak users, extra time slots are required to meet the objective because relaying operations are carried out serially.

NOMA, with its cooperative ability, is being explored in many fields. Even though it has been discovered that the geometric-based stochastic channel model (GBSM) offered better, more useful, and more realistic channel properties, the analysis of CR-NOMA with a mMIMO system is dependent on channel models, which are theoretically explained in a manner similar to the correlated-based stochastic channel model (CBSM). Research on GBSM channel models with the effective properties of CR-NOMA’s large antenna arrays and coding techniques has received little attention. As a result, it is crucial to investigate mMIMO CR-NOMA that considers channel features such as path loss and tilt angle. Further research into the coexistence of big antenna transmitters and coding methods is required [74]. In [75], the authors considered a shared DF relay for a cooperative relay sharing (CRS) network based on NOMA, which allowed two sources to connect with their associated users at the same time and frequency. Using a dynamic decoding order technique based on the max-min criteria, a new transmission idea was presented to reduce network outage probability at the expense of less complexity and overhead. The work also characterised outage probability expression for the proposed scheme. In [76], the effects of generic fading channel on cooperative NOMA networks with residual transceiver hardware impairments (RTHIs) was investigated. Imperfect CSI and SIC were considered. More specifically, the non-cooperative and cooperative scenarios were presented as two representative NOMA scenarios. For cooperative NOMA, it was shown that cooperative NOMA performed better than the non-cooperative NOMA in high SNR scenario as demonstrated by the authors findings in [76]. In [77], the effectiveness of a terrestrial satellite network consisting of a satellite as a source and its numerous terrestrial primary receivers and paired users placed on the ground, was examined. In this work, the nearest NOMA user operated in full-duplex (FD) mode, while the operation of the furthest NOMA user was performed with a decode-and-forward relaying approach. Importantly, the reasonable hypotheses of imperfect SIC based on NOMA and FD-based loop self interference were taken into account.

*Collaborative NOMA-aided relaying*: Collaborative NOMA-aided relaying (CNAR) was suggested in [78], and comprises of two NOMA connections referred to as relay-destination (R-D) and collaborative source-relay (S-R) connections. The relay message from the signal of S-R NOMA was extracted, and the power was modified to send the other portion to R-D-linked cell-edge users. The signals from the relay are sent in a specified frequency range in this method. The FD mode was utilised for the S-R and R-D connections to provide high throughput and support numerous users.

*HD-cooperative NOMA (CNOMA)/FD-CNOMA*: To address the power allocation issue and to optimize the possible user rate, the authors of [79] developed a hybrid HD/FD method for CNOMA. In addition, for numerous users, a relay selection mechanism was developed. Furthermore, NOMA-HD and FD executed, and allocated optimal power.

*FD-NOMA virtual pairing (VP)*: Using a relay, the nearest and furthest users with comparable gain are separated into different NOMA clusters. Over non-overlapping frequency bands, clustering was performed by utilising the VP technique between the nearest and several furthest users. The connectivity in BS and the farthest users is maintained through a specialised FD relay in FD-NOMA-VP, whereas the nearest user interacts to BS. In perfect interference cancellation, the FD-NOMA-VP system outperformed standard MA schemes by a factor of ten [80]. On the other hand, because of the rise in the influence of residual interference, performance suffers as interference cancellation is poor.

*NOMA power-line communication (PLC)*: PLC is a NOMA design proposed in [81] with two stages of power allocation. The BS sends data with differing power factors in the first stage, while the superposition-coded signal is received by both the relay and the destination. The signal is decoded at the destination with a greater power level while considering the other signal to be noise. The best power signal is first decoded at the relay and then canceled with the SIC to obtain the second symbol. The relay transmits the second symbol to the target in the second step, provided that it has been correctly decoded. The following are two advantages of utilising the NOMA in a PLC: (i) reduction in power transmitted at PLC modems, which alleviates the problem of compatibility affiliated with PLCs, and (ii) improved user fairness.

*Cooperative relaying selection (CRS)-NOMA*: The authors of [82] improved SE using multiplexed transmissions in the spatial method. The authors investigated the attainable rate for the best channel and found that CRS-NOMA performs better than the standard DF scheme.

*CRS-NOMA novel design (ND)*: This scheme was developed around a brand-new receiver design. In general, the source uses the superposition code to concurrently broadcast two signals, and the symbols are first decoded and then relayed with lesser allotted power using SIC [82]. However, in [83], signals either from an in-directed link or a directed link were decoded and then used the maximum-ratio combination and SIC were used to send the signal.

*N-best relay selection (BRS)*: This is an enhanced variant of CRS-NOMA in which many relays are utilised instead of just one [84]. The BRS scheme decides which relay can provide the best SNR or SINR at the destination to facilitate signal forwarding. The Rayleigh fading channel’s average rate was used to deduce the scheme’s performance.

*NOMA-RS/HD-NOMA-RS*: A relaying scheme with dual-hop capability is used in this design. A simultaneous interaction between sources was used in this approach to attain their goals. The relay in [85] sends a superposition coded composite sign using NOMA after receiving the transmitted symbols in parallel from both sources with exclusive allocated powers. In comparison to CRS-NOMA-ND and CRS-NOMA, the benefit of NOMA-RS is that it has numerous sources.

*FD-NOMA-RS*: FD-NOMA-RS is composed a of cooperative relay-based FD NOMA, with two source-destination pairs sharing the same full-duplex relaying (FDR) [86]. With a processing delay τ, FDR demultiplexes these symbols and concurrently sends an overlaid composite signal to the end destinations via downlink NOMA. The scheme’s performance was evaluated in terms of OP, ergodic sum capacity (ESC), and outage total capacity. They authors conducted an analytical analysis of their method by utilising both ideal and imperfect SIC circumstances to demonstrate its performance.

*Space-time block code (STBC)-NOMA*: A type of cooperative DF with a two-phase relaying method based on Alamouti STBC-NOMA [87] was considered. To investigate the system’s performance, the authors of [87] used the separate Rayleigh fading channels, and they also characterised the asymptotic approximations for ESC, OP, and outage SC. When compared to the standard CRS, it achieved substantial performance gains utilising NOMA and typical DF relaying methods.

*Dynamic relay selection (DRS)-fixed power allocation (FPA) and DRS-dynamic power allocation (DPA)*: For cooperative NOMA, these DF dual-relay selection methods employ distributive space-time coding [88]. In this method, the system power is fixed under the DRS-FPA scheme. One relay demultiplexes all users signals, and the optimal relay is chosen using the max-min criterion. The DF dual-relay selection technique using dynamic power allocation was utilised in DRS-DPA, with DPA being applied to both hops instead of only the second hop. The effectiveness of both approaches was assessed using a closed-form formulation of OP.

*Dynamic decode-and-forward (DDF) NOMA*: In this system, cooperative NOMA employs a DDF relay to improve anonymous users’ reception dependability. In DDF-NOMA [89], the user closest to the BS uses partial reception to demultiplex the overlaid signal combination received by users and then delivers the signal to the user who is the furthest away. Random user pairing was utilised to eliminate the requirement of channel status information at the BS. Randomly employed algorithms, and algorithms based on distance user-pairing are two user-pairing algorithms that require feedback and user location information, respectively [89].

*Two-way relay (TWR)-NOMA*: In TWR-NOMA systems, two distinct NOMA groups for communication used HD-DF to relay with each other [90]. The SINR of users was calculated using the effects of having perfect SIC and imperfect SIC. The authors also assessed the closed-form OP expressions in an asymptotic manner and an exact solution manner. The major goal of this system was to solve decoding order problems caused by perfect SIC.

*Buffer-aided (BA)-relay*: In BA-NOMA, transmission and reception of data packets through relays when the BS-R and R-D connections are disabled [91] boosts the system’s throughput by increasing the dependability of the relaying systems. Furthermore, the authors of [92] presented a one-source, two-user, buffer-aided relaying approach in which information was sent to two users via a dedicated relay.

*Multi-relay selection*: An AF and multi-relay selection approach was used to investigate the performance of cooperative NOMA by the authors of [93]. In this approach, the BS interacts with users through a chosen relay and establishes direct connections with them. The results demonstrate that as the relays quantity increases, so does the performance gain; however a constant performance is achieved with more than two relays in the better SNR area.

*Cooperative full-duplex relaying (CFR) NOMA*: Here, an incomplete SIC FD relaying system is used by the users close to the BS and functioned as an FD relays to facilitate relay to further users. This system was discussed in [94]. Under three different situations, the numerical results of this method were evaluated utilising parameters such as OP. The powers of BS and relay were fixed in the initial condition. The relay’s and BS’s power were optimised in the second condition to decrease the OP. Users’ fairness was considered, and the relay’s and BS’ power were tuned to maximise each user’s individual rate.

*NOMA-relaying broadcast channel (RBC)*: The analysis of cooperative SISO-NOMA relaying was investigated using a two-user model [95]. A compress-and-forward (CF) protocol was used by users close to the BS and functioned as an FD relay to facilitate relay to farther users in this approach. With noisy network coding (NNC) and CF relaying, its performance was evaluated by the attainable rate region of RBC. A dirty paper coding (DPC) was applied to the BS to improve its performance with respect to the acquired attainable rate region using CF and NNC. When compared to traditional NOMA, it produced superior outcomes.

**Figure 9:**
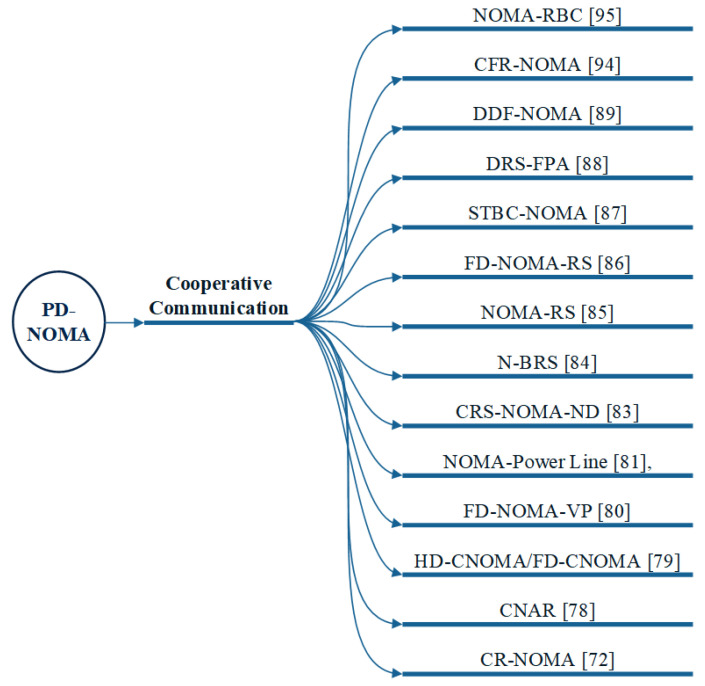
Cooperative communication in power domain NOMA and its types.

Types of cooperative communication in the current NOMA schemes are highlighted in Figure 9. Also, comparison of cooperative NOMA communication methods is provided in Table 3.

#### 3.2.2. NOMA with SWIPT Protocol

**Figure 10:**
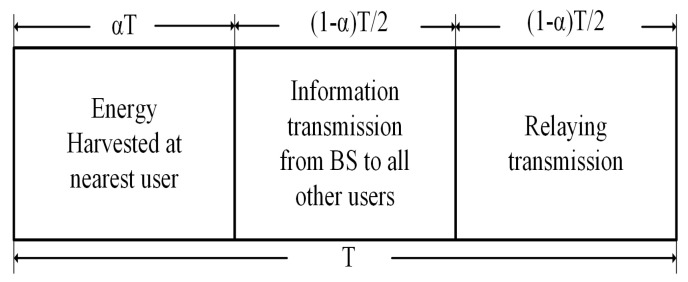
Time-switching (TS) power-splitting (PS) SWIPT Protocol in NOMA system.

Besides enhancing SE, energy efficiency (EE) is another significant issue in the 5G wireless networks that must be addressed. The majority of communication devices are supplied with batteries that have a limited lifetime. As a result, energy harvesting (EH) is a better approach for extending the lifetime of these energy-constrained systems as shown in Figure 10. EH methods initially gather energy from sunlight and wind as a renewable resources, but they are unreliable, because they are dependent on the environment. On the other hand, SWIPT is a common EH approach where energy is harvested in the decoding process. It collects energy even from interfering signals. When stronger users serve as relays for weaker users, their batteries drain quickly, according to C-NOMA. As a result, both strong and weak users’ signal strength is deteriorated. This challenge inspired academic and industrial researchers to combine SWIPT and C-NOMA to improve network EE in the following way.

*Cooperative (C)-SWIPT-NOMA*: NOMA utilising the SWIPT was originally investigated in [96], in which users were located randomly. In this approach, strong users collect energy from the BS’s RF signals during relaying and then forward the weaker users’ data. The authors of [97] built a C-NOMA transceiver with several antennas for one user and a single-antenna BS for the remaining users. At the transmitter side, a zero-forcing (ZF) beamforming architecture was used to analyse the C-SWIPT-NOMA performance with a BS and two users in [98]. Users that are close to the BS function as relays and have numerous antennas, whereas terminal users with a single antenna are farther away from the BS.

*C-SWIPT-MISO/SISO-NOMA*: In [99], the authors utilised the SWIPT-based MISO-NOMA system to implement the transmit antenna selection (TAS) scheme. Criterion-I and criterion-II were used to split the TAS scheme into two categories. They chose an antenna that delivers a channel with better fading conditions between the source and the far user and near user for each of these parameters. respectively. In contrast to [99], the authors of [100] used MISO and SISO methods to execute the C-SWIPT-NOMA transmission scheme. First and foremost, at the EH relays, a power splitting approach was utilized. The relays used this approach to forward information by solely collecting RF signals’ energy and limiting their battery usage. Second, the MISO approach was discussed in order to increase the data rate of users with strong channel conditions. Third, in order to explore practical applications, the SISO approach was utilised in the transmission strategy.

*NOMA-EH*: The authors of [94,101] integrated SWIPT-based NOMA, using EH protocol with relay nodes that enable communication between the BS and many users, similar to C-SWIPT-NOMA. More then one antenna was utilised on the BS and also at the users in this approach. At the BS and many users, TAS and maximum-ratio combining (MRC) methods were also employed. The performance of these methods was evaluated using a closed-form definition of outage across Nakagami-m fading and Rayleigh channel distributions.

*SWIPT-FD/CR-NOMA*: The authors in [102] looked at the effects of two different power distribution strategies in C-SWIPT-NOMA. The first method used fixed power, whereas the second used CR. Users’ power allocation coefficients were fixed under SWIPT-F-NOMA, and weak users were provided more power than strong users. The power allocation coefficients of SWIPT-CR-NOMA, on the other hand, were opportunistic. It offers a variety of reliability, complexity, and fairness trade-offs.

*SWIPT-NOMA-HETNET*: Having poor CSI, the authors in [103,104] used femtocells in a SWIPT-NOMA system for better resource allocation. Subchannel allocation was developed using the many-to-many matching approach. The macro-users’ preference lists were created based on the energy that they gathered from media broadcast satellite (MBS) RF transmissions.

#### 3.2.3. Hybrid NOMA

**Figure 11:**
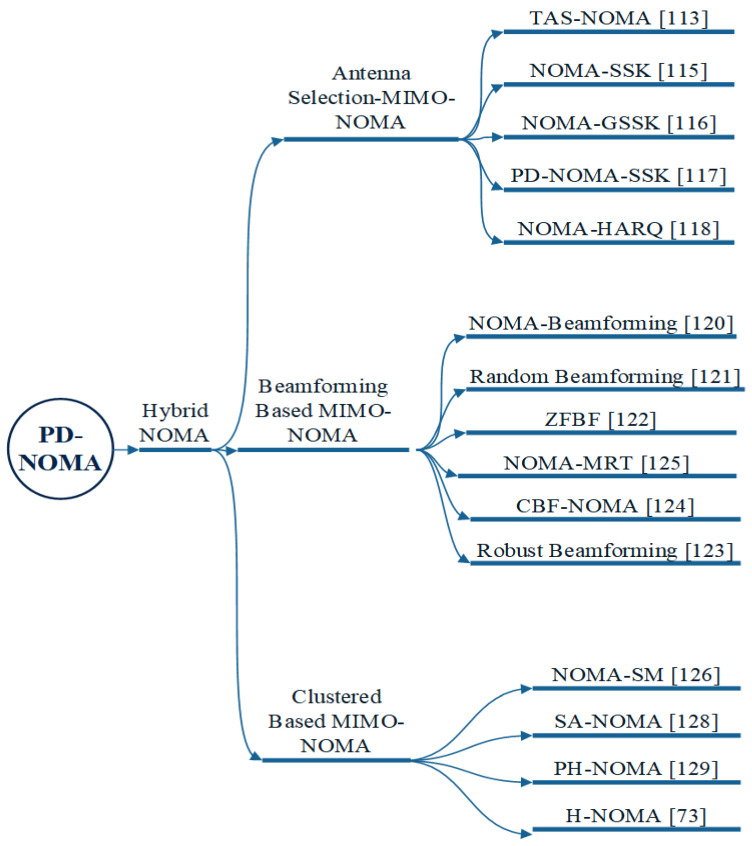
Types of hybrid NOMA in MIMO communication systems.

To improve the SE of sensors in IoT, PD-NOMA multiplexing, which is based on the hybrid TDMA method, was developed in [105]. By characterising the throughput and OP, the authors assessed the BackCom-assisted IoT network’s performance. Several types of Hybrid NOMA are classified in Figure 11.

*MISO and MIMO NOMA*: Currently, MISO-NOMA and MIMO-NOMA networks are widely used in 5G networks and have sparked a surge in academic and industrial interest. Systematic research on the development of NOMA in 5G networks began in 2014 [105,106]. Those efforts were focused on SISO systems to improve user fairness in the early stages of research. The flexibility of NOMA in serving numerous users utilising the same frequency-time resources is the reason for its adoption in SISO. Using NOMA in MISO and MIMO systems, on the other hand, improves spectrum reuse efficiency by adding diversity in the power domain. Signals from multiple users are mapped depending on the specified power value and then broadcast using the corresponding channel. Using the SIC technique, each user can identify the desired signals. MIMO-NOMA, in contrast to NOMA is a single-antenna system that uses high-dimensional power and beamforming [34] that introduces new features to power coefficient computation.

The benefits of MIMO-NOMA can be summarised as follow:

- *Spectrum efficiency*: MIMO-NOMA is able to minimise power consumption by utilising the power domain for user multiplexing. The SIC condition ensures that the received interference can meet the data rate requirement following the signal decoding. As power consumption decreases, more users may be supplied concurrently, improving spectrum efficiency.
- *Enhanced user cooperation*: This scheme can preserve service quality and fairness by adjusting power allocation between different types of users. By allocating more power to weak users, MIMO-NOMA may increase cell-edge quality and hence improve the cell-edge user effectiveness.
- *Many wireless transmission scenarios*: As the MIMO-NOMA scheme can easily be integrated with different modern MIMO technologies, such as cooperative multi-point (CoMP) technology [107] and cloud radio networks [108], MIMO-NOMA is often implemented as a cooperative method either inside a single base station (BS) or across numerous BSs.

Interference in MIMO systems lowers the network SE and QoS of the cell-edge user. Hence, NOMA is combined with MIMO, wherein the user must be careful with the allocation of the power in NOMA. A MIMO-NOMA comparison is provided in Table 4. As a result, in the MIMO-NOMA system, effective antenna selection, beamforming, and clustered based NOMA approaches are necessary.

*Back-scatter-aided NOMA*: Ambient back-scatter communication (ABC) and NOMA have recently been combined, and this latest combination has showed significant promise in connecting large-scale IoT in future unmanned aerial vehicle (UAV) networks. ABC’s main goal is to eliminate the need for batteries by harnessing the power of WiFi, TV towers, cellular base station, and UAV RF signals that are already in use. To modulate and reflect data between wireless devices, ABC uses smart sensor tags. On the other hand, NOMA enables simultaneous communication between multiple IoT devices [109]. The underlying concept of ABC is the potential for wireless device communication using currently existing ambient radio frequency waves. With better spectrum efficiency and extensive connectivity, NOMA has recently been studied extensively. In order to reduce the overall transmission power of ABC-NOMA cooperative vehicle-to-everything networks (V2XneT) while maintaining service quality, a new optimization approach was proposed in [110,111]. To be more precise, initially, the BS sends an overlaid signal to connected roadside units (RSUs). The RSUs then use the decode-and-forward protocol to deliver an overlaid signal to serving vehicles. The overlaid signal is also captured by a back-scatter device in the RSU coverage area, which modulates its own data to reflect them toward moving vehicles.

#### 3.2.4. Antenna Selection-Based MIMO-NOMA:

In this strategy, antenna selection helps to maintain a variety of a MIMO systems [112] in terms of capital and operational expenditure, complexity, and power consumption using numerous antennas at the same time. Researchers [112] have utilised the MIMO method in OMA systems, but owing to the inter-user interference, they were unable to obtain a significant gain when compared to MIMO-NOMA. As a result, the following alternatives are recommended to address this issue.

*TAS-NOMA*: The performance in terms of the sum-rate of a downlink MISO-NOMA system is explored in [113] using transmit antenna selection (TAS) at the BS, where the BS transmitter and each mobile user’s receiver have multiple antennae and a single antenna, respectively. Essentially, the finest antenna at the BS with the better SINR is chosen in the TAS-OMA method. A similar antenna at the BS that provides the largest sum rate is chosen in the TAS-NOMA system in [113]. In addition to applying an efficient TAS technique, in a two-user huge MIMO-NOMA system, a user-selecting technique is used to maximise the possible sum rate in [114] for two users with a single band and multiple users with multiple band cases.

*NOMA-space shift keying (SSK)*: This is a method that combines NOMA with SSK to elevate the SE of a cell-edge user. In contrast to typical modulation techniques, it is an approach that sends information using the antenna index [115]. SSK has several advantages, the most important of which is that it decreases transmitter overhead and receiver complexity. As a result, the employment of SSK in conjunction with NOMA is employed to elevate the SE of spatial modulation networks. This represents a promising technique for future wireless networks.

*NOMA-generalised (G)SSK*: This method is used to resolve the limitation of SSK in the transmit numbers of antennas. The authors of [116] proposed that NOMA-GSSK to elevates the SE of cell-edge users. Unlike NOMA-SSK, this concept has multiple transmit antennas. In contrast to MIMO-NOMA and NOMA-SSK, this system multiplexes the users in both the spatial and power domains, providing better EE and SE, and a low BER.

*PD-NOMA-SSK*: Because of the varying level of power that users require for operation in the PD-NOMA system, the amount of resources available for group communication is restricted. As a result, cryptographic keys are inefficiently dispersed between users. After evaluating this issue, the authors of [117] combined the SSK modulation method with PD-NOMA to meet the requirement for high SE. The authors further used MU-MIMO methods to multiplex PD-NOMA-SSK to improve system throughput.

*NOMA-hybrid automatic repeat request (HARQ)*: In [118], the authors investigated the single-user MIMO (SU-MIMO) system along with an HARQ design. This technique was suggested by the authors in order to eliminate inter-stream and inter-user interference. The effects of precoding matrix adaptation, user pairing, and transmission power assignment (TPA) ratio adaptation on retransmission in MIMO-NOMA systems was also investigated. The study demonstrated that NOMA has a considerably greater HARQ probability than OFDMA, and that variable retransmission techniques could improve the performance.

#### 3.2.5. MIMO-NOMA with Beamforming

Multi-cast beamforming is yet another method used in MIMO-NOMA to better systems’ total capacity, particularly in the event of many users. Specifically, there are single-beam and multi-beam methods. All users in a group receive the same beam in the single-beam method, but in multiple-beams, different beams are provided to different user groups [119].

*NOMA-beamforming*: The authors in [120] investigated the beamforming approach in a DL scenario for a MIMO-NOMA with a multiple-user system. In this method, a user pair with differing channel characteristics share the same beam. To decrease interference, the authors suggested a user clustering and power distribution method. This approach maximised the system’s total capacity. In this technique, a fairly minor signal was sent to the weaker users (because of bad channel characteristics), while both lower and higher priority signals were sent to the strong users closet to the BS.

*Random beamforming NOMA*: On the BS side, the authors of [121] addressed the application of the random beamforming approach. In this method, each user in a cluster receives a single beam with the same coefficient as the power allocation. The authors further suggested a spatial filter to minimise inter-beam and inter-cluster interference. Furthermore, the authors used the idea of reuse of fractional frequency to enhance power distribution across many beams.

*Zero-forcing beamforming (ZFBF)*: This method reduces inter-cluster interference in varied channel conditions. To reduce interference and achieve maximum performance, the authors of [122] presented a dynamic power allocation and user clustering method. Moreover, these authors used the ZFBF approach in a downlink scenario for a multi-user MIMO-NOMA system.

*Robust beamforming NOMA*: It is notable that a majority of researchers discussed beamforming design in NOMA systems with the perfect CSI knowledge. However, given the quantization and estimation issues of the channel, it is difficult for the BS to determine the users’ desired CSI. In [123], a resilient beamforming approach based on a framework for worst-case optimization was investigated to solve the problem of norm-bound channel uncertainty in downlink multi-user-MISO-NOMA systems. For users within a cluster, a single beamformer was utilised in this method.

*Coordinated beamforming (CBF) NOMA*: The authors of [124] examined the issue of interference in MIMO-NOMA system. The interference alignment CBF (IA-CBF) and interference channel alignment CBF (ICA-CBF) algorithms were suggested by the authors. Two BSs cooperate with each other with respect to their beamforming vectors in these systems to improve the QoS of cell-edge users without exchanging data across cells. Inter-cluster and inter-cell interference are eliminated in both of these methods, while intra-cluster interference is mitigated by NOMA’s SIC approach.

*NOMA-maximum ratio transmission (MRT)*: The authors of [125] looked at the development of information broadcasting that switches to MMSE beamforming and NOMA-MRT to keep the sum rate constant. The goal of this technique was to obtain the highest possible sum rate of the system. The authors also claimed that, with its correlated and fully aligned channel vectors, MMSE beamforming achieves a rate that is almost comparable to NOMA-MRT. With an orthogonal channel, however, the MMSE beamforming outperforms the NOMA-MRT.

#### 3.2.6. Clustered-Based MIMO-NOMA

The clustered-based users and the proper beams for each cluster are designed using clustered-based MIMO-NOMA (CB-MIMO-NOMA). Through the use of an appropriate transmit detector and precoder, CB-MIMO-NOMA efficiently reduces inter-cluster interference. This method ensures that the beam connected with a certain cluster is orthogonal to other clusters users. The disparity between the users’ channel conditions grows as a result of cluster isolation. The SIC method of NOMA may also be used to reduce intra-cluster interference.

*NOMA spatial modulation (SM)*: SM is an energy-efficient method in which the information bits are sent using an index of the transmit antennas and amplitude-phase modulation. In [126], the authors combined NOMA and SM in a downlink MU-MIMO situation. Inter-user interference may be minimised using this system by using the SIC. Furthermore, the authors of [126] presented an optimal power allocation scheme analysed on the basis of symbol rate errors to obtain high SE.

**Table 4:** Comparison of NOMA based on MIMO.

NOMA Variant	Problem Discussed	Metric	Advantages	Open Issues
TAS-NOMA [127]	Multiple antennas at transmitter	SR	Reduces the cost, complexity and power	Imperfect CSI
NOMA-SSK [115]	Low SE of cell-edge users	EE BER SE	Reduces decoding complexity	Power allocation scheme
NOMA-GSSK [116]	Low SE of cell-edge users	EE BER SE	Reduces computa-tional complexity	SIC analysis
PD-NOMA-SSK [117]	Secerecy	BER SE	Improves network throughput	Power allocation problem
NOMA-HARQ [118]	Inaccurate MCS selection	SE		Extension of MU-MIMO
NOMA-BF [120]	Inter-cluster, inter-user interference	SR	Improves QoS	Imperfect CSI
Random-BF-NOMA [121]	Inter-cluster, inter-beam interference	Th	Reduces CSI feedback	Analysis of imperfect SIC
ZFBF-NOMA [122]	Inter-cluster interference	SE Th	Maximises overall throughput	Multicell scenario
ROBUST-BF-NOMA [123]	Inter-beam, inter-cluster interference	SR	Maximises worst case sum rate	Extension of MU-MIMO
C-BF-NOMA [124]	Inter-cell, inter-cluster interference	Th	Increases throughput of cell-edge users	Imperfect SIC
NOMA-MRT [125]	Sum rate	SR	Maximises weighted sum rate	Extension of mMIMO
NOMA-SM [126]	Inter-user interference	SE EE	Enhanced SE	Imperfect CSI
SA-NOMA [128]	Inter-cluster interference	OP	Provides large diversity gain	Imperfect CSI and SIC
PH-NOMA [129]	Inter and intra-cluster interference	SR OP	Minimises total power consumption	MIMO-NOMA scenario
H-NOMA [73]	Transmission power	OP	Reduces the transmission power	

*Signal alignment (SA)-NOMA*: In this technique, interference between clusters is removed in the MIMO-NOMA system. The authors of [128] used this approach to investigate the OP of downlink and uplink MIMO-NOMA broadcasts in a single-cell setting. Different QoS criteria were met using the fixed power allocation (FPA) approach, but the CR-inspired power allocation technique ensures the higher QoS requirements of users.

*Hybrid (H)-NOMA*: In comparison to ZFBF, H-NOMA is a low-complexity precoding algorithm. It depends on the concept of the quasi-degradation idea and may be used in real-world networks. A MISO-NOMA system with two users was investigated in [73] under the sequential user pairing technique. The authors used average transmit power and OP to numerically and analytically assess H-NOMA’s performance.

*Projection Hybrid (PH)-NOMA*: In [129], PH-NOMA was proposed to reduce intra- and inter-cluster interference by combining H-NOMA precoding and the traditional ZFBF method. The features of quasi-degradation hlwere used to combine PH-NOMA with a pairing algorithm with inversion and projection to achieve minimal complexity and reduce overall interference. The diversity and OP gain were used to evaluate the performance of these methods.

#### 3.2.7. Reconfigurable Intelligent Surface (RIS)-Aided NOMA

Intelligent reflecting surfaces are well accepted as possible physical 6G wireless platform supporter. With their capacity to alter the nature of interacting electromagnetic waves by carefully controlling the phase alterations of reflections, RISs have shown promise for increasing the SE of RIS-NOMA. RISs can be implemented to increase channel gains and ensure excellent network performance. The property of NOMA to provide greater user fairness and RIS properties of best channel gain can be theoretically utilised in combination to ensure that all devices receive the same data rate. Significant worries about the power consumption, energy efficiency, and the coverage area of future networks have been raised as a result of the rising demands for higher data rates and better wireless communications. NOMA networks supported by the RIS-related work is presented in this subsection. Some significant RIS-NOMA systems discussed in literature are given in Table 5.

*Throughput in RIS-NOMA*: The optimization approach introduced in [130] is frequently used to alternatively establish the phase shifts of RIS and the transmit beamforming at the BSs because of the optimization problems non-convexity defined for improved throughput. The authors of [131] initially showed that phase shift changes are not required for uplink and downlink NOMA systems, which helps to solve the issue and improves the signalling overhead, when considering a wireless-powered RIS-NOMA system with several devices/users. In [132], beamforming at the BS and phase shifts at the RIS were optimised in addition to assigning the channel and the NOMA users decoding order to increase the system throughput. The RIS system’s sum rate was investigated in [133] in order to evaluate the NOMA, FDMA, and TDMA approaches. Additionally, in RIS-NOMA systems ([134,135]), the utilization of a BS with multiple antennas was taken into consideration. Plans for optimising the active and passive beamforming at both the BS and RIS were then discussed in order to increase the sum rate.

*RIS-NOMA in mmWave and massive MIMO communications*: Millimeterwave [136], massive MIMO [137], and other major 6G communication system technologies are all very compatible with RIS-NOMA. In order to maximise the system sum rate, Zuo et al. [136] considered the architecture of mmWave networks and presented an optimization approach in which the power distribution and reflection coefficients were alternatively tuned. The outcomes displayed in [136] demonstrate the value of RIS integration in mmWave-NOMA systems. Leveraging upon the fact that mmWave massive MIMO systems were created owing to the narrow wavelength of mmWave transmissions which allows for the deployment of many antennas, the unique approach has shown potential of mitigating the effects of imperfect SIC and take advantage of polarization diversity as a result. The use of RIS-NOMA with mmWave and mMIMO systems was taken into consideration by Liu et al. [137], where the antenna power and leakage were concurrently explored. Regarding imperfect SIC in NOMA, the extent to which RISs can control wave polarization in dual-polarized MIMO-NOMA networks was explored in [138].

*RIS-NOMA in THz Communications*: Terahertz communications are discussed as a possible enabling influence for achieving ultra-high information rates and providing a sufficient transmission capacity for 6G wireless networks. In order to serve applications that call for accurate localization and environment sensing, future wireless communication networks will use higher frequencies [139]. Due to the restricted number of propagation channels available for such high frequencies (mostly because of high penetration losses, high path loss values, and low scattering), sensing and localization accuracy may suffer. An effective way to address this issue, particularly in NLOS communication circumstances, is by creating a smart radio environment utilising RIS [140]. According to [141], employing NOMA transmission results in greater average data rates for situations in which users are spread out over a wide range (distance) of access points, demonstrating that NOMA is a convincing approach for THz networks. In the literature, the RIS-assisted THz network is examined [142]. In addition to these investigations, there is need for research into the RIS-assisted NOMA transmission terahertz communication network.

*Power minimization in RIS-NOMA*: Power efficiency and energy efficiency (EE) are crucial measures for assessing the future effectiveness of wireless communication networks. The performance of NOMA and OMA for transmit power minimization was examined in [143], which offers a crucial rule for user pairing in RIS-aided systems with multiple users and resource blocks. In [144], a multi-cluster MISO RIS-NOMA system was investigated with the goal of reducing the overall transmit power. This paper proposes an optimization approach based on second-order cone programming (SOCP), beamforming, and phase shift to reduce the BS’s overall transmit power. In contrast to single-RIS-assisted NOMA networks [145], a multi-RIS and multi-cluster NOMA network was examined in [146]. It was suggested that the transmit power at the BS can be minimised by jointly optimising the beamforming at the BS, power distribution to the NOMA users, and RIS phase shifts.

*Energy efficiency (EE) maximization in RIS-NOMA*: Another crucial indicator for future green communication networks is EE. The ratio of information bits provided to energy consumed is known as the EE. The goal of EE maximization is to obtain the best trade-off between a better sum rate and less power, which is distinct from transmit power minimization and sum-rate maximization and creates a fractional programming challenge. The EE maximization issue was investigated in RIS-NOMA networks based on two-users models [147]. Driven by the advantages of RIS-NOMA, beamforming optimizations based on sequential convex approximation (SCA) and phase shift optimization algorithm based on SDR were suggested for maximization of EE. Additionally, by allocating subchannels based on matching theory, difference-of-convex (DC) programming can be used to maximise the EE of NOMA systems [148].

**Table 5:** Selected significant RIS-NOMA systems discussed in literature.

RIS-NOMA Variant	Problem Discussed	Variables	Results	Open Issues
**Throughput and** **Data Rate** **Maximization**	SIS-RIS-NOMA [149]	Power, phase shift	Weighted sum rate performance	Optimal PACs
	MISO-RIS-NOMA [134]	Phase shift, active beamforming	Maximises system sum rate	Additional powertransmission
	mmWave aided-RIS-NOMA [136]	Beam selection, active beamforming	Provides a near-optimal solution	Resource allocation scheme
	Massive MIMO-RIS-NOMA [138]	Phase shift of RIS	Dual-polarised RISs	Channel estimation with limited feedback
**Power Minimization**	SISO-RIS-NOMA [143]	Beamforming at BS	User pairing in RIS-NOMA	Incremental redundancy
	MISO-RIS-NOMA [150]	Phase shift of RISs, beamforming	Minimises transmit power	Multiple antennas at receiver
**EE Maximization**	MISO-RIS-NOMA [147]	Phase shift of RISs, beamforming	Maximises system energy efficiency	Limited CSI knowledge
**Physical Layer Security (PLS)**	Multi-user-RIS-NOMA [151]	Passive beamforming	Quality of channel	Secrecy rate
**Channel Estimation**	RIS-NOMA-assisted multi-user comms [152]	Limited pilot symbols	Good estimation of channel	Imperfect SIC
**THz Communication**	RIS-NOMA assisted THz comms [153]	Average data rate	Best transmission capacity	Limited CSI knowledge

*Physical layer security (PLS) in RIS-NOMA*: RIS is also critical in NOMA networks for enhancing privacy and security concerns in a network due to the potential to autonomously change the wireless propagation environment. Ensuring a good secrecy rate in a multi-user NOMA scenario, where the reliability of some of the legitimate users’ channels from a transmitter is inferior to the wiretap channel is a challenging task [154]. This encourages the use of RISs in NOMA to improve of physical layer security [155]. Theoretically, the direct and reflected pathways can be added at the user and subtracted at the eavesdropper by placing RIS close to the authorised NOMA users or eavesdropper and effectively developing the passive beamforming [156]. Therefore, a positive secrecy rate is attained in RIS-NOMA networks by reducing the eavesdropper capability of signal reception and improving the legitimate signal reception capability. Knowing eavesdropper’s CSI is crucial for effective beamforming design, which is almost impossible to do, to enable RIS-NOMA secure communication. In [151], the authors examined secure transmission in an RIS-NOMA network while considering a real-world eavesdropping scenario with an inadequate eavesdropper CSI.

*Channel estimation in RIS-NOMA*: It is crucial to have the CSI, because NOMA employs SIC to detect the signals. In order to simplify the implementation of SIC’s ability to identify the signal at the receiver, it is assumed that the majority of NOMA-related applications have perfect CSI. There exists only a few studies in the literature that concentrate on NOMA applications with imperfect CSI [157,158,159]. The huge RIS reflecting elements, their unique hardware limitations, and the multi-user aspect of NOMA make channel estimation in RIS-NOMA systems is a complex challenge. On the basis of RIS connection with devices, there are two types of RIS channel estimation algorithms that have been proposed in the literature: semi-passive and completely passive. Devices (such as inexpensive sensors) are embedded into the RIS’s reflective components in semi-passive sensing. Therefore, signals from the BS or users to the RIS channels can be calculated depending on their received pilots by allowing the BS and each user to transmit pilot signals. For instance, in [160] a new reflection pattern at the RIS was devised for OFDMs to help the access point in estimate a channel based on the user-received pilot signals. Similar to this, Ref. [161] proposed a unique pilot-based channel estimate framework using a limited pilot symbols for an RIS-assisted multi-user communication system.

#### 3.2.8. Machine Learning (ML)-Based NOMA Communications

It is unfeasible to design the number of variables that need to be configured for NOMA-enabled future wireless systems as the complexity of the system model increases. Due to the combinatorial complexity, it is challenging to jointly optimise the many design variables. Therefore, there is a lot of potential for machine learning (ML) to be used in resolving the optimization challenges for NOMA systems that are covered in this study. The applicability of ML and deep learning (DL) for resource optimization issues in IoT and other cellular networks is explored in [162], along with a brief overview of the NOMA systems applications. Next, we deliberate on NOMA combined with ML.

*Rate optimization in ML based NOMA*: The most popular strategy in the NOMA literature for dealing with the challenge of combinatorial complexity is to divide it into a number of smaller problems. As necessary, one or more of these sub-problems can be helped using ML approaches. For instance, the user selection sub-problem is solved in [163,164] using an unsupervised clustering approach, while the power allocation issue is dealt with using traditional optimization. The channel assignment is dealt with using a DRL algorithm in [165], which studies a multi-carrier environment, while the power allocation is again dealt with using traditional optimization. Power optimization can also use ML techniques when more variables are introduced to the problem. For instance, the authors of [166] allocated power using an RL algorithm when a deliberate jammer was present. The key point is that ML techniques are capable of handling a portion of these issues and can be utilised in conjunction with conventional methods for optimization when the model complexity increases and introduces more variables, as it is typical for NOMA-enabled systems to have more designed variables for NOMA systems such that the numbers of designed variables becomes too large for ML algorithms as complexity is added via multi-cell and carrier, cooperative, etc. In such circumstances, a deep learning neural network as suggested in [167], for example, may be investigated to determine the parameters that have the greatest influence on the sum-rate performance. Following that, the chosen variables to be optimised are selected using traditional optimization methods or alternative ML techniques.

*Quantum ML*: While using ML in NOMA-enabled systems has many appealing benefits, there are drawbacks as well. The amount of computing power needed to perform some of these ML algorithms is one of the main issues with ML algorithms. To help with this, new trends such as quantum machine learning (QML) are studied for future-generation networks. The authors of [168] focused on how QML may greatly improve the problems in multi-objective optimization that require the adjustment of many constraints and their parameters, which is a typical scenario for NOMA. The huge amount of data needed to apply ML algorithms presents another problem. Today, communications networks gather a lot of data which are of no use, including CSI, user positions, and other information, using them solely for immediate scheduling decisions. These can be used in ML algorithms and then in NOMA-enabled systems, as big data processing develops quickly.

*Supervised ML*: The channel changes so quickly when ML is applied at the physical layer, so there is less time for ML algorithms to gather useful data to learn from. This presents another issue. This makes it particularly difficult to deploy supervised algorithms that gain knowledge from the past at the physical layer. Supervised learning techniques, however, have been investigated for estimation and feedback of the channel, MIMO detection, and other associated issues in mMIMO systems [169,170].

In what follows, we pay particular attention to the ML methods of unsupervised learning, reinforcement learning, and deep learning. These are used to solve rate optimization issues in the NOMA-based system models examined in this study. These elaborations present a number of potential directions for further research into the use of ML in NOMA-enabled systems.

*Unsupervised ML*: Unsupervised machine learning techniques do not use historical training data. Because of the sub-problem of selecting different users, clustering techniques in particular are suitable for NOMA-based systems. As we previously noted, the standard method in NOMA-related work is to partition the problem into numerous sub-problems because of the combinatorial difficulty of the combined optimization of a significant number of design variables. One of the first sub-problems that academics typically address is user clustering or user pairing. This sub-problem can be solved using clustering algorithms such as K-means clustering. The authors of [163] investigated a mmWave-NOMA system and made efficient use of K-means clustering by taking advantage [164] of the high correlation between user channels and the dominance of the LoS path in mmWave propagation. K-means clustering is useful in system models where an LoS path dominates because it simplifies the task of locating spatially connected users.

*Deep Learning (DL)*: Deep learning (DL), which incorporates numerous layers and may collect the features from the source data before executing tasks such as classification, is a more powerful type of machine learning [171]. It is challenging to put a DL method into practice in NOMA-enabled systems due to the fast-changing nature of the physical channel. In NOMA-based systems, deep learning capabilities can still be used in a number of different ways. In [172], a deep recurrent neural network was built to quickly deliver convergence with less computational complexity resource allocation solutions for the NOMA heterogeneous IoT. When the number of objectives and variables becomes significantly high, a neural networks such as those explained by the authors of [167] are effective for obtaining crucial factors. However, when combined with reinforcement learning, using deep learning to optimise the rate for NOMA systems shows the most promise, because the agent uses a multi-layered neural network to make judgments in a practical environment. Deep reinforcement learning (DRL) is the name given to this method to emphasize the integration of DL and RL, and is discussed next.

*Deep reinforcement learning (DRL)*: In next-generation wireless communications systems, DRL algorithms are studied with respect to a number of resource allocation issues [173]. These concepts are readily applicable to the NOMA systems examined in this study. In [165,166], DRL agents can be utilised in NOMA systems for allocating the power level and assigning their corresponding channels. A BS, an end user, or a UAV can be a DRL agent. These nodes must be able to make decisions on their own based on interactions with other nodes in the system. DRL algorithms are also capable of optimising the sum rate of sub-problems, similar to how ML clustering was used for the selection of users sub-problems. Relay and spectrum selection in relay-based NOMA networks and in UAV-NOMA networks are directions using DRL that are possibly worthy of exploration. In this approach, DRL can be used to supplement NOMA-specific optimization algorithms in the NOMA networks. For systems using a hybrid NOMA and MU-MIMO method, such as [174], an RL algorithm can be utilised to solve another interesting problem.

*Online learning*: To solve the issue of NOMA network flexibility, i.e., adopting a new user without having much overhead, online learning is a crucial class of ML algorithms. For instance, the authors of [163] created a web-based ML clustering technique that can accommodate additional users up to a predetermined threshold.

## 4. Open Issues and Challenges

**Figure 12:**
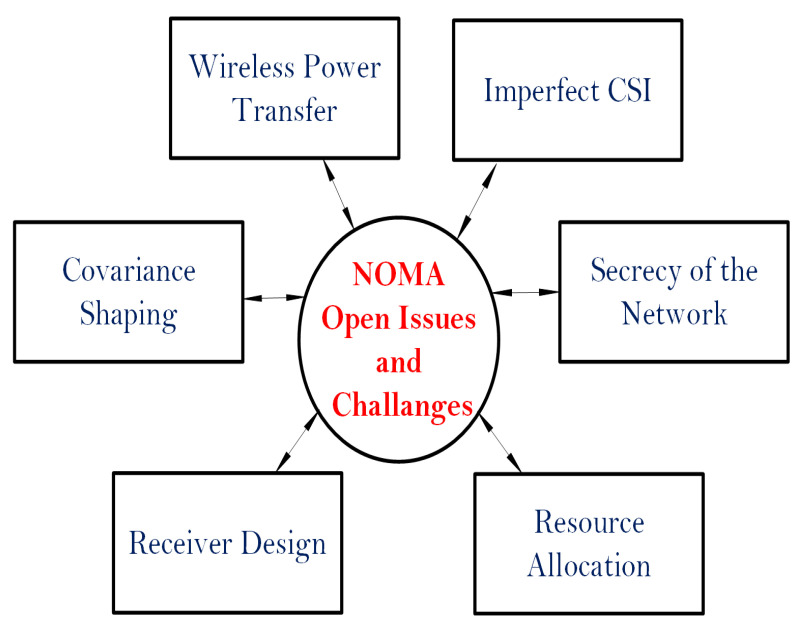
Some of the open issues and challenges in cooperative PD-NOMA.

The usage of NOMA in the context of 5G and B5G is in its early stages. To realise its influence on end users, a number of outstanding concerns and research challenges must be addressed. This section uses NOMA variations to explore the present and future communication problems given in Figure 12.

### 4.1. Wireless Power Transfer

This method extends the operational lifetime of power-constrained devices [175,176,177,178]. Solar, thermoelectric, and wind effects are used in energy harvesting; however, these are not practical, because they are dependent on the time of day, the location, and the setting. This challenge has been handled by harvesting RF signals by allowing battery-constrained systems to maintain their energy.

The initial focus of wireless power transfer (WPT) has been on high-power and long-distance transmission; however, the continued development has been constrained by the possible reduction in transmission efficiency and associated health risks brought on by high-power applications. Alternative wireless information and power transmission techniques have proven to be crucial not only for theoretical study but also for the operational cost reduction and the sustainable expansion of wireless communications due to the massive energy consumption growth with the ever-increasing number of linked devices. The distance between the base station and the device in a communication network is important for both information and power transfer [179]. Therefore, more advancements in far-field WPT techniques are required.

Implementing NOMA with wireless power transfer for IoT applications using SWIPT protocol must be solved in order to enhance network EE, as discussed in Section 3.2.2.

### 4.2. Imperfect CSI

The case where the BS has perfect knowledge of the CSI has been the focus of majority of research on NOMA systems. However, it can be difficult to achieve the perfect CSI in reality. Several authors working on NOMA systems have addressed a significant amount of work with optimal CSI for resource allocation and identification of multiple users [180,181,182,183]. The evaluation of NOMA networks with imperfect CSI is particularly essential because the quality of CSI has a significant impact on the encoding and decoding of NOMA. To deal with the model with imperfect CSI, ideal joint precoders are required to eliminate the interfering signals from the BS. Given the preceding explanation, it is an open problem that must be handled in order to decrease the impact of errors.

In terms of CSI, there are two types: instantaneous CSI and statistical CSI. The present channel conditions are known in instantaneous CSI (or short-term CSI), which can be compared to the impulse response of a digital filter. This allows the sent signal to be adapted to the impulse response, allowing the received signal to be optimised for spatial multiplexing or low bit error rates. The authors of [182,183,184] analyzed instantaneous signal strength with imperfect CSI in NOMA systems. Statistical CSI (or long-term CSI) denotes that the channel’s statistical characterization is known. The type of fading distribution, the average channel gain, the line-of-sight component, and the spatial correlation can all be included in this description. This information, similar to instantaneous CSI, may be utilised to optimise transmission. The authors of [185,186,187,188] analysed statistical CSI-based NOMA systems.

### 4.3. Covariance Shaping

The covariance shaping approach can be achieved by using statistical beamforming, which allows users to successfully excite a sufficient fraction of all possible propagation paths between themselves and the BS. We show that our strategy works well for restoring orthogonality between users that are too near to one other. As a result, operations such as pilot decontamination and BS beamforming can benefit from this technique [189,190,191]. It entails applying statistical beamforming to each UE ahead of time during the downlink data transmission phase to ensure signal subspace separation between users that would otherwise be substantially overlapping. As a result, covariance shaping may be used for both pilot decontamination and statistical precoding. It offers the distinct advantage of converting the non-Kronecker nature of large channels into a benefit. Covariance shaping has not been utilised in NOMA systems previously and remains an open issue.

### 4.4. Secrecy of the Network

NOMA technology is receiving an increasing amount of attention as a result of its various benefits, including high data rates, improved spectrum and energy efficiency, broad connectivity, and reduced latency. However, because wireless channels are typically open, secure data transfer in wireless communication systems continues to be a major difficulty. Physical layer security (PLS) techniques have recently been introduced to improve the stability of NOMA systems and address wireless transmission problems. Users who are closer to the channel are typically given lower power levels than users who are farther away and have worse channel conditions. In order to obtain their own signals, near users will therefore need to block the high-power transmissions (signals of far users). To facilitate the effective deployment of NOMA, these major security issues must be resolved [192]. The problem of protecting NOMA utilising a physical layer has recently been the focus of various studies in the literature. Because it depends on the randomness and dynamics of the physical layer and uses straightforward operations to guarantee the security of communicated data, PLS is a novel security approach that has proven to be more effective and robust than existing upper-layer security schemes [193].

Secrecy of the network is an essential problem that must be addressed in each wireless communication generation [178,194,195,196,197]. Because it is subject to eavesdropping, a signal sent using a wireless channel requires special attention. Security remains an unresolved question for the NOMA method, particularly in the cases of MIMO and mMIMO. In wireless networks, providing secure communication has always been a priority. Traditionally, security has been handled at the protocol stack’s top levels, using encryption methods that are unaffected by the physical features of wireless channels [198] However, with the rapid expansion in the number of low-complexity, low-power, and computationally challenged devices in 5G and IoT networks, the notion of PLS is gaining traction. PLS uses the unpredictability of wireless channels to ensure that a hostile eavesdropper cannot decipher the sent data [199].

### 4.5. Resource Allocation

NOMA gives users with lower channel gains greater control in order to maintain user fairness. It is not practicable to apply NOMA to all users at once due to the increased system overhead for channel feedback coordination and error propagation. As a result, the concept of user pairing (UP) has come into existence [200], where users in the cell are separated into several clusters and NOMA is used inside each cluster. A NOMA system’s performance is heavily reliant on both UP and power allocation (PA). Common names for them include resource allocation (RA). The goal of the RA in NOMA is to choose which users will be paired and how much power will be provided to each user within each cluster. Although it is computationally demanding, an exhaustive search of all potential user pairings and transmit power allocations can be used to achieve the best performance of NOMA RA. Additionally, if dynamic UP and PA are used, additional signaling overheads are introduced due to the decoding order in SIC and PA ratios. Furthermore, it is utilised to allocate users with radio resources, resulting in increased user fairness, capacity, data rate, and EE [34,201,202,203]. Because the spectrum has limited radio resources, resource distribution to consumers becomes challenging when a multi-cell has a large number of users. Because of its capacity to serve multiple users at varying power levels at the same time, NOMA has made effective use of its resources. Because of cross-channel and co-channel interference, however, utilising the NOMA approach to allocate resources to users is still an open issue for the researchers.

### 4.6. Receiver’s Complexity

The core philosophy of NOMA is to offer all users the entire available bandwidth in order to maximise bandwidth utilization. However, this solution involves a rise in receiver complexity because superposition coding is necessary to generate the adequate signal, and then, the original information (data) is retrieved using successive interference cancellation and decryption techniques on that signal [204]. Even if they have the worst channel gains, every user in the cluster must decode data from every other user. Due to the receiver’s increased complexity, there has been an increase in energy consumption as well. The performance gain of NOMA can be enhanced by using SIC at cell-edge users, as demonstrated by the authors of [205]. The development of an easy-to-use, effective SIC receiver is essential to NOMA. Multi-stage SIC lowers multi-path fading and bit error rate. Also, performance of the system is impacted by the signal’s decoding order. High-SNR signals are initially deciphered. The performance of the SIC receiver is enhanced by a low-complexity, highly effective power allocation algorithm [206]. A practical implementation of this is its use to decode and detect desirable signals when non-idealities and imperfections cause error propagation in SIC. Due to the signal processing required for SIC, receiver complexity increases as the number of UEs rises.

A user’s performance is impaired by the SIC receiver’s complexity and error propagation [207,208,209,210]. As a result, non-linear detection that is more efficient with better performance is necessary for addressing this problem and decreasing the impact of error propagation. To increase signal detection performance, the data symbols are decoded, demodulated, and exchanged by receivers more effectively. However, concerns including the propagation of errors, effective design of the receiver, and signal identification accuracy must be alleviated to improve receiver performance.

### 4.7. NOMA in Mobile Edge Computing (MEC)

IoT devices typically have limited memory and processing power. In order to sustain an IoT-enabled network, some of the more complex application tasks of tremendous interest can be managed at a central location. There are still numerous difficulties in transferring to the MEC servers. The first is that many IoT devices may use the same MEC servers, making it difficult to guarantee that these servers have the computational capabilities necessary to complete operations quickly. Second, the battery life of IoT devices is limited. Task computation locally on the device and data transfer to a MEC server both require energy and may cause the battery to discharge quickly. To ensure timely completion of the computing activities and efficient use of the batteries of the IoT devices, real-time applications require the optimization of resource allocation in MEC-based networks. A resource allocation issue that maximises downlink sum rate of NOMA users has been put forth in [211]. Later, Ref. [211] has evaluated an adaptation that jointly optimises the resource allocation and power allocation. Because NOMA permits several users to access the same subcarrier concurrently, using NOMA results in a greater system-weighted sum-rate performance in both works than identical systems that use the OMA scheme. Hence, NOMA in MEC is one of the future research challenges in academia and industry.

### 4.8. NOMA in Intelligent Reflecting Surfaces (IRS)

IRSs’ capacity to align the channel vectors of the user has been demonstrated to be a key facilitator of NOMA cluster formation. IRS phase shift gives an extra level of flexibility for NOMA cluster users, similar to BF techniques in MIMO-NOMA networks, and are employed to organise user clusters that are served by PD-NOMA techniques. The active BF at the BS and at the IRS passive BF have been optimised in two recent studies [134,212] to obtain the greatest sum rate for the system’s users and show an improvement over OMA-IRS systems. A new optimization method for improving spectral efficiency in a multi-cell RIS-NOMA network with signal decoding faults was presented in [213]. In particular, the BS’s power budget and NOMA users’ transmit power are optimised in each cell at the same time as the reflection matrix of the RIS. The joint problem is defined as non-convex because of the restrictions on objective function and quality of service, making it extremely difficult to determine the best overall solution.

By combining NOMA and IRS to create IRSNOMA systems, it is possible to increase NOMA cluster formation potential via active and passive BF approaches. The more complex user orders and additional constraints from the passive reflectors that must be taken into account in rate optimization problems present new design challenges.

### 4.9. NOMA with Imperfect SIC

The near user has a perfect and complete understanding of the signal information relating to the far user in an ideal SIC procedure. In contrast, in imperfect SIC circumstances, this information is not fully available. As a result, in this imperfect SIC, there appears to be some residual inter-user interference. The interference from the far user cannot entirely be eliminated by the near user [214]. Numerous factors, including hardware limitations, finite length codes, and channel estimation errors, might contribute to the imperfect SIC. The performance of NOMA systems and optimization problems are significantly impacted by the imperfect SIC. In particular, it is difficult to attain reliable concavity conditions for the optimization problem of ideal power allocation (i.e., without power order constraints) in multiple-carrier NOMA systems when the SIC is imperfect [215]. In comparison to the perfect SIC, the imperfect SIC has a larger outage probability and a lower ergodic capacity [216]. The SIC procedure carried out in NOMA systems with relays may result in problems that would cause an outage. Both relay nodes and destination nodes may experience the defective SIC, which can also cause an outage [217,218].

## 5. Conclusions

This article provided a complete assessment of the literature, as well as an updated evaluation of NOMA-related work on cooperative communication with major network-allowing mechanisms in 5G and B5G networks. Based on the studies presented, it appears that research in this topic is still in its early stages, leaving the field wide open for future investigation. Integrated networks must satisfy stated criteria such as reliability, efficiency, and higher system performance in emerging technologies. This article also covered an overview of NOMA, a state-of-the-art technology that enhances spectrum efficiency, energy efficiency, outage probability, cell-edge transmission rate, and latency. NOMA’s performance and advantages over OMA are also highlighted. Furthermore, the literature on hybrid NOMA, a comparison of cooperative communication-based existing NOMA schemes, and a comparison of NOMA based on MIMO are discussed. Lastly, open research challenges are highlighted.

## Figures and Tables

**Table 1:** A comprehensive list of existing survey papers list that addressed the PD-NOMA and its comparison with our work.

Type	[23]	[26]	[40]	[41]	[42]	[43]	Our Work
Physical layer security	✔	✘	✘	✔	✔	✘	✔
MIMO and mMIMO	✔	✔	✔	✔	✔	✔	✔
ComP	✘	✔	✘	✘	✘	✘	✔
Cooperative Network	✔	✔	✔	✔	✔	✔	✔
mmWave	✘	✔	✘	✔	✔	✔	✔
MEC	✘	✔	✘	✔	✘	✘	✔
THz	✘	✔	✘	✔	✘	✘	✔
IRS	✘	✔	✘	✔	✘	✘	✔
ML	✘	✔	✘	✔	✘	✘	✔
Clustered NOMA	✘	✘	✘	✘	✘	✘	✔
Hybrid NOMA	✔	✔	✔	✔	✘	✘	✔
TAS	✔	✘	✘	✘	✘	✘	✔
Covariance shaping	✘	✘	✘	✘	✘	✘	✔
Channel estimation	✘	✘	✘	✘	✘	✘	✔

**Table 2:** Types of NOMA.

NOMA	Domain Multiplexing	Received Type	Advantage	Open Issues
LDS [60,61,62,63,64,65,66]	Spreading codes	MPA	Without CSI	redundancy in coding issue
SCMA [67,68]	Codebooks	MPA	Without CSI	Difficult detection
PDMA [70]	Pattern	SIC/MPA	Better performance	Less overload
IDMA [71]	Interleaver	Signal estimation	High user overload	High latency
PD-NOMA [72,73]	Power	SIC	Less receiver complexity	Low user overloading

**Table 3:** Cooperative communication based existing NOMA schemes.

NOMA Variant	Problem Discussed	Performance Metric	Advantages	Open Issues
C-NOMA [72]	QoS of far user	Ergodic capacity outage probability	Reduces the system complexity, maximises diversity gain,	Multiple antennas at BS, optimal PACs
CNAR [78]	Multiple cell-edge users	Ergodic capacity outage probability	High throughput, better data rate	NOMA interference on cell-edge
FD/HD-CNOMA [79]	Power allocations	User rate	Better user rate	Resource allocation scheme
FD-NOMA-VP [80]	Interference cancellation	Ergodic capacity outage probability	Comparably better results	Additional power transmission with MIMO systems
NOMA-PLC [81]	Average capacity	Sum capacity	Compatible with PLC, two symbols forward	AF protocol, FD systems
CRS-NOMA [82]	Spatially multiplexing	Average rate	Improves average power	SIC stability Channel estimation with limited feedback MIMO systems
CRS-NOMA-ND [83]	Receiver design	Ergodic capacity outage probability	Better ergodic sum rate, better MRC scheme results	Incremental redundancy
N-BRS [84]	Rate gain	Average rate	Reduce complexity	Best relay position
NOMA-RS [85]	SIC	Sum capacity	Serves a large number of users	Evaluates outage
FD-NOMA-RS [86]	Spectral efficiency	Ergodic capacity outage probability	Serves a large number of users	With MIMO-NOMA
STBC-NOMA [87]	Reliability	Sum capacity	Boosts the spectral efficiency	Multiple antennas at receiver
DRS-FPA-NOMA [88]	Diversity gain	outage probability	Better reception reliability	Nakagami-m fading channel
DDF-NOMA [89]	Reception reliability	Outage probability	Better reception reliability	Has limited CSI knowledge
CFR-NOMA [94]	Imperfect SIC	Ergodic capacity outage probability	Better fairness, maximises achievable rate	Channel allocation scheme
NOMA-RBC [95]	Full duplex relaying	Achievable throughput	Improves weak user rate	Extended to MU-MIMO

## Nomenclature

Acronym	Description	Acronym	Description
1G	First generation	G-RQ	Generalized Rayleigh quotient
2G	Second generation	GSM	Global system for mobile
3G	Third generation	HD	Half-duplex
4G	Fourth generation	HetNet	Heterogeneous network
5G	Fifth generation	H-NOMA	Hybrid NOMA
AF	Amplify and forward	ICA	Interference channel alignment
AR	Augmented reality	IQF	Indefinite quadratic form
BA	Buffer aided	JD	Joint-diagonalization
BER	Bit error rate	LDS	Low-density spreading
BRS	Best relay selection	LoS	Line-of-sight
BS	Base station	M2M	Machine-to-machine
CA	Carrier aggregation	MA	Multiple access
CBF	Coordinated beamforming	MISO	Multiple-input single-output
CCI	Co-channel interference	mmWave	Millimeter wave
CDF	Cumulative distribution function	MPA	Message passing algorithm
CDI	Channel distribution information	MRC	Maximum ratio combining
CDMA	Code division multiple access	MRT	Maximum ratio transmission
CEEs	Channel estimation errors	NNC	Noisy network coding
CF	Compress-and-forward	OP	Outage probability
CFR	Cooperative full-duplex relaying	PACs	Power allocation coefficients
CoMP	Coordinated multipoint	PAPR	Peak-to-average-power ratio
CR	Cooperative relaying	PD	Power domain
C-RAN	Cloud radio access network	PDF	Probability density function
CRS	Cooperative relay selection	PH	Projection hybrid
DDF	Dynamic decode and forward	PLC	Power line communication
DF	Decode-and-forward	PLS	Physical layer security
DL	Downlink	QoS	Quality of service
DPC	Dirty parity coding	QPSK	Quadrature phase-shift keying
DRS	Dynamic relay selection	RBC	Relaying broadcast channel
EE	Energy efficiency	R-D	Relay-to-destination
EH	Energy harvesting	RF	Radio frequency
eMBB	Enhanced mobile broadband	RHIs	Residual hardware impairments
EMC	Electromagnetic compatibility	SA	Signal alignment
ESC	Ergodic sum capacity	SC	Superposition coding
FD	Full-duplex	SCMA	Sparse code multiple access
FDD	Frequency-division duplex	SINR	Signal-to-interference-plus-noise ratio
FDR	Full duplex relaying	SLNR	Signal-to-leakage-plus-noise ratio
FFR	Fractional frequency reuse	SM	Spatial modulation
FPA	Fixed power allocation	ZFBF	Zero-forcing beamforming
